# A common genetic factor for Parkinson disease in ethnic Chinese population in Taiwan

**DOI:** 10.1186/1471-2377-6-47

**Published:** 2006-12-22

**Authors:** Hon-Chung Fung, Chiung-Mei Chen, John Hardy, Andrew B Singleton, Yih-Ru Wu

**Affiliations:** 1 Department of Neurology, Chang Gung Memorial Hospital and College of Medicine, Chang Gung University, 199 Tung-Hwa North Road, Taipei, 10591, Taiwan; 2 Laboratory of Neurogenetics, National Institute on Aging, Porter Neuroscience Building, 35, Convent Drive, Bethesda, MD20892, USA; 3 Reta Lila Weston Institute of Neurological Studies, University College London, 1 WakefieldStreet, London, WC1 N1PJ, UK; 4 Molecular Genetics Unit, National Institute on Aging, National Institutes of Health, Bethesda, MD 20892, USA

## Abstract

**Background:**

Parkinson's disease (PD) is the most common neurodegenerative movement disorder, characterized clinically by resting tremor, bradykinesia, postural instability and rigidity. The prevalence of PD is approximately 2% of the population over 65 years of age and 1.7 million PD patients (age ≥ 55 years) live in China. Recently, a common *LRRK2 *variant Gly2385Arg was reported in ethnic Chinese PD population in Taiwan. We analyzed the frequency of this variant in our independent PD case-control population of Han Chinese from Taiwan.

**Methods:**

305 patients and 176 genetically unrelated healthy controls were examined by neurologists and the diagnosis of PD was based on the published criteria. The region of interest was amplified with standard polymerase chain reaction (PCR). PCR fragments then were directly sequenced in both forward and reverse directions. Differences in genotype frequencies between groups were assessed by the *X*^2 ^test, while *X*^2 ^analysis was used to test for the Hardy-Weinberg equilibrium.

**Results:**

Of the 305 patients screened we identified 27 (9%) with heterozygous G2385R variant. This mutation was only found in 1 (0.5%) in our healthy control samples (odds ratio = 16.99, 95% CI: 2.29 to 126.21, p = 0.0002). Sequencing of the entire open reading frame of LRRK2 in G2385R carriers revealed no other variants.

**Conclusion:**

These data suggest that the G2385R variant contributes significantly to the etiology of PD in ethnic Han Chinese individuals. With consideration of the enormous and expanding aging Chinese population in mainland China and in Taiwan, this variant is probably the most common known genetic factor for PD worldwide.

## Background

Parkinson's disease (PD) is the most common neurodegenerative movement disorder, characterized clinically by resting tremor, bradykinesia, postural instability and rigidity. The prevalence of PD is approximately 2% of the population over 65 years of age and 1.7 million PD patients (age ≥ 55 years) live in China [1,2]. Mutations in five genes have been shown to cause parkinsonism: α-synuclein (dominant), parkin (recessive), *DJ-1 *(recessive), *PINK1 *(recessive) and *LRRK2 *(dominant) [3]. Since *LRRK2 *mutation has been reported as a cause of PD, a number of *LRRK2 *substitutions have been identified to be pathogenic [4]. To date, the most common genetic variant underlying sporadic PD is the G2019S variant reported at up to 40% frequencies in different Caucasian populations [5]. The genotype-phenotype correlations have been studies on this mutation [6]. The clinical manifestations of the G2019S carriers closely resemble those of typical PD with pleomorphic pathological findings [7,8]. The rarity of the G2019S mutation in Chinese population suggests the occurrence of this mutation resulted from a common European founder [9]. Recently, a common variant Gly2385Arg was reported in ethnic Chinese PD population in Taiwan [10,11]. In this study, we analyzed the frequency of this variant in our independent PD case-control population of Han Chinese from Taiwan and explore the genotype-phenotype correlations of this prevalent alteration in our Taiwanese PD cohort.

## Methods

### Subjects

305 patients with sporadic idiopathic PD (46% Female, Mean age of onset: 61.8 ± 10.6 years, Range: 30–91 years) were selected from the neurology clinic of Chang-Gung Memorial Hospital. 176 unrelated healthy adult volunteers (46% female, Mean Age at exam: 59.6 ± 9.6 years, Range: 38–86 years) were matched for ethnic origin and area of residence. These normal control individuals are genetically unrelated to the individuals in the patient group. All patients were examined by a neurologist (YR Wu or CM Chen), and the diagnosis of PD was based on the published criteria [12]. Patients with evidence of secondary parkinsonism or with atypical features such as early dementia, ophthalmoplegia, early autonomic failure, and pyramidal signs were not included in this study. All examinations were performed after obtaining informed consent under a protocol approved by the internal ethics and scientific boards of National Institutes of Health.

### Genotyping and statistical analysis

Genomic DNA was extracted from peripheral blood using standard protocols. The region of interest was polymerase chain reaction (PCR)-amplified as previously described. Assay of the G7153A polymorphism in all the individuals was performed by PCR amplification with primer pair 5'-ATGCAGCTTTCAGTGATTCCA-3' (forward) and 5'-TCTGAAAAGATGGTGCTGAGAAG-3' (reverse). Cycle sequencing in forward and reverse directions was performed on purified PCR products with the Big Dye Terminator chemistry and run on an ABI 3100 automated DNA sequencer. (Applied Biosystems, Foster City, CA). Differences in genotype frequencies between groups were assessed by the *X*^2 ^test. *X*^2 ^analysis was used to test for the Hardy-Weinberg equilibrium (HWE).

## Results

Of the 305 patients screened we identified 27 (9%) with heterozygous for the G2385R variant. This mutation was only found in 1 (0.5%) in our healthy control samples (odds ratio = 16.99, 95% CI: 2.29 to 126.21, p = 0.0002). In both groups, there were no deviations from HWE at this G7153A locus. None of the patients with the 2385R variant had a family history of PD. Age of onset for the 2385R carriers was 33–79 years (mean 61.5 ± 11.3 years). Meanwhile, age of onset of 285 PD cases without the 2385R variant was 31–91 years (mean 61.9 ± 10.5 years). Sequencing of the entire open reading frame and flanking intronic sequence (>50 bp) of LRRK2 in 2385R carriers revealed no other coding variants.

The clinical presentations of all G2385R carriers showed no difference from typical idiopathic PD with definite asymmetric onset, bradykinesia, resting tremor (4–6 Hz), rigidity (Table 1). All carriers showed response to dopamine replacement therapy based on clinical observation.

## Discussion

*LRRK2*, encodes a protein called dardarin that is postulated as a member of the ROCO protein family. There are five conserved domains in this protein. The *LRRK2 *kinase domain belongs to the MAPKKK subfamily of kinases. The active sites of all kinases are located in a cleft between an N-terminal and a C-terminal lobe, typically covered by an activation loop in an inactive confirmation. The residue G2385 substitution lies in the WD40 domain. WD40 domain is speculated to be capable of of mediating protein-protein interactions [13]. Hypothetically, *LRRK*2 G2385R variant may cause a conformational change of the WD40 domain of *LRRK2*. The functional effects of the G2385R substitution on dardarin have yet to be established.

The lack of the G2385 variant in other populations, except ethnic Chinese PD populations, highlights the fact that the contribution of different genetic factors varies amongst different populations [11]. Furthermore, these discrepancies between mutation frequencies across different ethnicities worldwide lend support to the hypothesis of a founder effect. Our findings delineate *LRRK2*-related disease as a common form of sporadic PD in the Chinese population.

Based on our results, the G2385R variant contributes significantly to the etiology of PD in ethnic Han Chinese individuals. With consideration of the enormous and expanding aging Chinese population in mainland China and in Taiwan, this genetic variant is probably the most common known genetic risk factor for PD worldwide. A routine testing for this mutation at least in our population may be cost-effective. Though whether our finding extends to other Asian populations needs further verification. Complete analysis of the entire 51 exons of *LRRK2 *will be warranted before recommendations and guidelines for screening of *LRRK2 *in other ethnic PD patients for diagnostic purposes and genetic counselling be implemented globally.

## Conclusion

In conclusion, our data suggest that the G2385R variant contributes significantly to the etiology of PD in ethnic Han Chinese individuals. With consideration of the enormous and expanding aging Chinese population in mainland China and in Taiwan, this variant is probably the most common known genetic factor for PD worldwide.

## Competing interests

The author(s) declare that they have no competing interests.

## Authors' contributions

HCF carried out the molecular genetic studies participated in the sequence alignment and drafted the manuscript. CMC and YRW participate in the design of the study and collection of data. JH participated in the study, and participated in its design and coordination. ABS conceived of the study and a critical review of the manuscript. All authors read and approved the final manuscript.

## Pre-publication history

The pre-publication history for this paper can be accessed here:



## Figures and Tables

**Table 1:** Comparison of clinical manifestations between PD 2385R carriers and PD non 2385 carriers

	**PD (2385R Carrier)**	**PD(Non 2385R Carrier)**
Number of subject	27	278
Age of Onset(Mean ± SD)	61.5 ± 11.3	61.9 ± 10.5
Resting tremor (n, %)	26(96)	252 (88)
Bradykinesis (n, %)	27(100)	269 (97)
Rigidity (n, %)	26(96)	261(94)
Asymmetrical onset (n, %)	20(74)	203(73)

PD: Parkinson disease

## References

[B1] Rocca WA (2005). Prevalence of Parkinson's disease in China. Lancet Neurol.

[B2] Zhang ZX, Anderson DW, Huang JB, Li H, Hong X, Wei J, Yang EL, Maraganore DM (2003). Prevalence of Parkinson's disease and related disorders in the elderly population of greater Beijing, China. Mov Disord.

[B3] Cookson MR, Xiromerisiou G, Singleton A (2005). How genetics research in Parkinson's disease is enhancing understanding of the common idiopathic forms of the disease. Curr Opin Neurol.

[B4] Farrer M, Stone J, Mata IF, Lincoln S, Kachergus J, Hulihan M, Strain KJ, Maraganore DM (2005). LRRK2 mutations in Parkinson disease. Neurology.

[B5] Mata IF, Kachergus JM, Taylor JP, Lincoln S, Aasly J, Lynch T, Hulihan MM, Cobb SA, Wu RM, Lu CS, Lahoz C, Wszolek ZK, Farrer MJ (2005). Lrrk2 pathogenic substitutions in Parkinson's disease. Neurogenetics.

[B6] Hernandez D, Paisan RC, Crawley A, Malkani R, Werner J, Gwinn-Hardy K, Dickson D, Wavrant DF, Hardy J, Singleton A (2005). The dardarin G 2019 S mutation is a common cause of Parkinson's disease but not other neurodegenerative diseases. Neurosci Lett.

[B7] Paisan-Ruiz C, Jain S, Evans EW, Gilks WP, Simon J, van der BM, Lopez M, Aparicio S, Gil AM, Khan N, Johnson J, Martinez JR, Nicholl D, Carrera IM, Pena AS, de Silva R, Lees A, Marti-Masso JF, Perez-Tur J, Wood NW, Singleton AB (2004). Cloning of the gene containing mutations that cause PARK8-linked Parkinson's disease. Neuron.

[B8] Zimprich A, Biskup S, Leitner P, Lichtner P, Farrer M, Lincoln S, Kachergus J, Hulihan M, Uitti RJ, Calne DB, Stoessl AJ, Pfeiffer RF, Patenge N, Carbajal IC, Vieregge P, Asmus F, Muller-Myhsok B, Dickson DW, Meitinger T, Strom TM, Wszolek ZK, Gasser T (2004). Mutations in LRRK2 cause autosomal-dominant parkinsonism with pleomorphic pathology. Neuron.

[B9] Fung HC, Chen CM, Hardy J, Hernandez D, Singleton A, Wu YR (2006). Lack of G2019S LRRK2 mutation in a cohort of Taiwanese with sporadic Parkinson's disease. Mov Disord.

[B10] Tan EK, Zhao Y, Skipper L, Tan MG, Di Fonzo A, Sun L, Fook-Chong S, Tang S, Chua E, Yuen Y, Tan L, Pavanni R, Wong MC, Kolatkar P, Lu CS, Bonifati V, Liu JJ (2006). The LRRK2 Gly2385Arg variant is associated with Parkinson's disease: genetic and functional evidence. Hum Genet.

[B11] Di Fonzo A, Wu-Chou YH, Lu CS, van Doeselaar M, Simons EJ, Rohe CF, Chang HC, Chen RS, Weng YH, Vanacore N, Breedveld GJ, Oostra BA, Bonifati V (2006). A common missense variant in the LRRK2 gene, Gly2385Arg, associated with Parkinson's disease risk in Taiwan. Neurogenetics.

[B12] Hughes AJ, Daniel SE, Kilford L, Lees AJ (1992). Accuracy of clinical diagnosis of idiopathic Parkinson's disease: a clinico-pathological study of 100 cases. J Neurol Neurosurg Psychiatry.

[B13] Greggio E, Jain S, Kingsbury A, Bandopadhyay R, Lewis P, Kaganovich A, van der Brug MP, Beilina A, Blackinton J, Thomas KJ, Ahmad R, Miller DW, Kesavapany S, Singleton A, Lees A, Harvey RJ, Harvey K, Cookson MR (2006). Kinase activity is required for the toxic effects of mutant LRRK2/dardarin. Neurobiol Dis.

